# The symbiotic bacteria *Frischella perrara* in honey bees mitigate varroa mite infection

**DOI:** 10.1128/spectrum.00960-26

**Published:** 2026-06-15

**Authors:** Hongfang Wang, Xueying Niu, Li Lei, Tong Zhou, Ge Zhang, Baohua Xu

**Affiliations:** 1 College of Animal Science and Technology, Shandong Agricultural University, Tai’an, China; 2 Key Laboratory of Efficient Utilization of Non-Grain Feed Resources, Shandong Agricultural University, Tai’an, China; 3 Shandong Provincial Key Laboratory of Animal Nutrition and Efficient Feeding, Shandong Agricultural University, Tai’an, China; University of Minnesota Twin Cities, St. Paul, Minnesota, USA

**Keywords:** gut microflora, honey bee, *Frischella perrara*, *Varroa destructor*, hygiene behavior, olfaction

## Abstract

Honey bees are vulnerable to various pathogens and pests, and ectoparasitic *Varroa destructor* has become the main cause of population decline of western honey bees (*Apis mellifera*) worldwide. Currently, chemical acaricides are the primary methods for controlling Varro*a* mites (*V. destructor*), and developing non-chemical control technique is urgently needed for beekeeping industry. We aimed to evaluate if the symbiont gut bacteria from bee colonies were attributed to Varroa tolerance and could be developed as a control agent. We sequenced 16S rDNA of gut microbiota between surviving colonies at a non-acaricide treated apiary (i.e., anti-mite colonies, AMCs) and conventional mite-susceptibility colonies (CMSCs) at an apiary with routine acaricide treatment. We found that AMCs harbor higher abundance of *Frischella*. By supplementing CMSCs with *F. perrara* in the field, we found decreased Varroa infestation intensity with more fallen phoretic Varroa and more injured pupae to be removed (an indicator of hygienic behavior). By supplementing honey bees with *F. perrara* in laboratory, we found increased proboscis extension reflex frequency and expression of odor-binding proteins in their antennae (OBP 11, 12, 16, and 21), indicating a potential molecular mechanism of enhanced hygienic behavior. This study suggests a novel function of the gut symbiont *F. perrara* in mitigating Varroa parasitism, implying its potential to serve as a new method to control Varroa mites through enhancing bees’ olfactory sensitivity.

**IMPORTANCE:**

The parasitic mite of honey bees, *Varroa destructor*, is the major challenge of beekeeping industry across the world. We propose a practical control method, applying a gut symbiont bacterium (*F. perrara*) to bee colonies to address this global challenge without the side effects of using chemical treatment methods. We found *F. perrara* treatment can lead to a higher number of fallen mites from adult bees’ body. The potential mechanism is to improve honey bees’ hygienic behavior to clean mites through promoting their olfactory sensitivity, increasing the chances to detect mites. Varroa control by administrating *F. perrara* does not harm honey bee health and avoid product contamination and pesticide resistance to mites. This method may provide a sustainable Varroa control tool in realistic beekeeping industry.

## INTRODUCTION

The widespread ectoparasitic Varroa mite (*V. destructor*) has become the most severe pest of western honey bees (*A. mellifera*) across the world. The original host of Varroa mite was eastern honey bees (*Apis cerana*), and this pest was transferred to western honey bees when it was introduced into Asia, ultimately causing spread throughout the world (1). Eastern honey bees have developed high tolerance to Varroa mites through intensive hygienic behavior during the long co-evolution history, making this pest less lethal. However, western honey bees have not developed hygienic behavior as strong as eastern honey bees to mitigate Varroa mite damage in the short co-evolutionary history (2). Without acaricidal treatments, western honey bee colonies can hardly survive beyond 2–3 years (3, 4). Beekeepers usually rely on chemical acaricides to control Varroa mites to prevent colony failure; however, acaricide use reduces Varroa pressure on honey bee colonies, slowing down the development of Varroa resistance. In contrast, acaricides can make Varroa rapidly develop resistance to acaricides (5). At the same time, acaricide use causes drug residues in colony components and bee products, affecting bee health and food safety.

In addition to Varroa control through acaricides, discovering honey bees with increased tolerance to Varroa mite and breeding these Varroa-tolerant linages is a common, friendly approach for sustainable Varroa control. Western honey bees have shown the development of Varroa tolerance across the world, including specific regions in Europe, Africa, and North America and South America (6–11). The most common mechanisms of tolerance of honey bees to Varroa mites include more active grooming and hygienic behaviors of adult bees (10, 12–14) and adaption of larvae or pupae through suppression of mite reproduction (15).

Gut bacterial symbionts have been found to increase honey bees’ tolerance to Varroa; however, the previous studies only cover limited number of bacteria species (16–18). And, it remains unclear how those bacteria regulate honey bees’ behavior to enhance tolerance to Varroa at the molecular level. Honey bees harbor a gut bacterial community comprising about eight major phylotypes, including *Gilliamella apicola* (Gamma-1), *F. perrara* (Gamma-2), *Snodgrassella alvi* (Beta), *Bartonella apis* (Alpha-1), *Commensalibacter* (Alpha 2.1), *Parasaccharibacter* and *Bombella* (Alpha 2.2), *Bifidobacterium* and *Lactobacillus* (Firm-4, Firm-5) (19–21). Those core phylotypes maintain nutrition digestion and absorption and parasite defense (16, 22, 23). Parasitic infestation intensity (ectoparasitic *V. destructor* and endoparasitic *Nosema apis* and *Nosema ceranae*) affects honey bee gut bacterial community (24, 25). And conversely, gut bacteria may have potential to change parasitic infestation intensity. For example, the bacteria, *Lactobacillus johnsonii* CRL1647 isolated from the intestinal tract of worker bees, has been shown to lower the infestation intensity of Varroa mites (26). In addition, the bacteria residing on honey bees’ cuticle, including *Lactobacillus kunkeei*, *Bacillus thuringiensis*, and *Bifidobacterium asteroids,* cause 95%–100% mite mortality within 3 days (27). Those studies indicated a potential role of honey bee symbiotic bacteria as natural antagonists of Varroa mites.

By investigating the gut symbionts of surviving colonies from our acaricide-free apiary and colonies from acaracide-treated colonies, our study will answer three questions: (i) if gut bacterial community of untreated surviving bees differ from Varroa-susceptible bees using 16S rDNA sequencing, and which key taxa drive the bacteria community change; (ii) if supplementing Varroa-susceptible colonies with a bacterium being found in higher abundance in untreated surviving colonies in our study can enhance their tolerance to Varroa; and (iii) how this key taxa may enhance honey bees’ Varroa tolerance through changing hygienic gene expression. By evaluating the effects of the symbiont bacteria on enhancing honey bees’ tolerance to Varroa and clarifying the underlying mechanisms, we aim to suggest a sustainable pest control strategy to resolve Varroa challenge for beekeeping industry.

## MATERIALS AND METHODS

### Characterization of Varroa infection rate and gut bacteria community of untreated surviving colonies

#### Honey bee colony management and Varroa infection rate assessment

Honey bee colonies (*Apis mellifera* Ligustica) used in this study were from two apiaries (named as A and B) 5 km apart and were both originally from sister queens. During 2021 and 2022, apiary A did not apply acaricides, while apiary B applied acaricides (twice a year in May and September). Other feeding and management conditions were similar between two apiaries. After 2 years (i.e., in 2023), six colonies survived and showed normal colony population and vitality. All six untreated surviving colonies from apiary A are named as anti-mite colonies (AMC), and five colonies from apiaries B with routine treatments are named as conventional mite-susceptible colonies (CMSC).

To test if the surviving colonies from apiary A had increased tolerance to Varroa mite than Apiary B, the infection rate of Varroa mites of all six surviving colonies from apiary A and five colonies from apiary B was determined in August in 2023 (28). Three hundred adult honey bees were randomly selected from each colony and placed into a large jar. Ethanol (75%) was added to the jar to fully submerge the bees, and the jar was shaken for 20 s to dislodge phoretic mites (28). Bees and ethanol were then poured over a 6-mesh sieve (aperture: 3.35 mm) to separate the bees and mites, while a second jar was positioned beneath the sieve to collect the ethanol with the dislodged mites. The ethanol from the second jar was subsequently poured over a 40-mesh sieve (aperture: 0.38 mm) to obtain mites. This procedure was repeated until no mites were detected on the 40-mesh sieve in three consecutive trials. The number of bees and the number of mites (NOM) were counted to calculate the mite infection rate in adult honey bees using the following equation. Above procedure was repeated three times for each colony, and the average of the three measurements was used as the mite infection rate calculation.


$$Themitesinfectionratesinadultbees=\frac{NOM}{300}\times 100\%$$

### Adult bee sampling for genomic sequencing

Honey bee guts were collected from 7-day-old adult bees of six AMCs and five CMSCs to compare gut bacteria community by sequencing 16S rDNA. The queen of each colony was first confined on an empty comb for 5 h using a queen spawning controller (Changge Jihong Beekeeping Equipment Co., He’nan, China) to lay eggs. The comb with laid eggs was then transferred to the top hive box, and the queen was kept in the bottom hive box, with a queen excluder between two boxes. When the confined brood grew to the pupal stage, the sealed brood comb was located in a meshed cage (Changge Jihong Beekeeping Equipment Co., He’nan, China) which can separate the newly emerged worker bees from other bees. At the 21st day, the newly emerged worker bees (1 day old) were labeled by painting their thorax using Yu’s method (29). The labeled bees were released back to corresponding hives and collected at the seven day (i.e., 7 days old). The 30 bees at 7 days old were then washed in 75% ethanol and kept on ice before dissection. The entire intestine of 30 individual bees was dissected and combined into a pooled sample for extracting gut bacterial DNA under aseptic conditions (30).

### Gut microflora DNA extraction and 16S rDNA sequencing

The gut microbial DNA was extracted using QIAamp 96 PowerFecal QIAcube HT kit (QIAGEN, Germany) according to the manufacturer’s protocols. The DNA concentration, purity, and integrity were verified using a NanoDrop 2000 (Thermo, USA) (the ratio of A260/A280 being between 1.8 and 2.0) and 1% agarose gels. The full length of 16S rDNA of the ribosomal RNA genes was amplified via PCR using primers 27F (5′- AGRGTTTGATYNTGGCTCAG-3′) and 1492R (5′-TASGGHTACCTTGTTASGACTT-3′). Amplified products were extracted from 2% agarose gels and purified according to the manufacturer’s instructions using the AxyPrep DNA Gel Extraction Kit (Axygen Biosciences, Union City, CA, USA). The purified DNA was then quantified using the ABI Step One Plus Real-Time PCR System (Life Technologies, Foster City, CA, USA). The SMRT Bell Template Prep Kit (PacBio) was used for damage repair, terminal repair, and joint connection of the PCR products, which were purified and recovered by AMpure PB beads to obtain library.

Sequencing was performed on PacBio Sequel II platform. Circular consensus sequencing (CCS) was obtained using the smrtlink tool provided by Pacbio. Raw CCS sequences were then preprocessed using q2-dada2-CCS software to detect and cut off the adapter. After trimming, the qualified raw CCS sequences were further filtered and denoised, and the chimera reads were cutoff to obtain the representative reads and the ASV abundance table. The representative read of each ASV was selected using QIIME2 package. All representative reads were annotated and blasted against Silva database (Version 138) using q2-feature-classifier with the default parameters.

### Assessment of the mite-tolerance property of susceptible bees fed with *F. perrara* in the field

Ten conventional mite-susceptible colonies of equal population were randomly divided into two groups, with five colonies in each group. One group was fed a 50% sucrose solution, serving as the control group; while, the other group was administered 1 × 10^6^ cfu/mL solution of *F. perrara* (Leibniz Institute DSMZ) in 50% sucrose at a dosage of 300 mL every 2 days for 6 months. *F. perrara* was directly mixed with 50% sucrose before feeding. All other management practice remained consistent between two groups.

### The mite infection rates in adult bees and the proportion of the fallen mites

The mite infection rate (MIRA) in adult bees was measured using the same method in the Characterization of Varroa infection rate and gut bacteria community of untreated surviving colonies section at a frequency of once a week in August. White acrylic sheets coated with Vaseline were placed at the bottom of the hives for a consecutive 7-day period to collect fallen Varroa mites (FV). The sheets were collected on the seventh day and immediately replaced with new ones for the next collection. Two rounds of assessment of fallen mites were conducted. During the same time, the colony population (CP) of adult bees were assessed. Each frame in each colony was photographed, and the area covered by adult bees on the surface of the frame was measured using ImageJ software. If a frame was completely covered, the population was recorded as 1 frame of bees; if half of the frame was covered, it was recorded as 0.5 frame. The proportion of dropped mites due to grooming (PDMG), as an indicator of assessing grooming behavior, was calculated using the following equation. The average of PDMG in two assessment trials from each colony was used for statistical analysis.


$$ThePDMG=\frac{ThenumbersofFV}{CP*MIRA}\times 100\%$$

### The mite infection rates in sealed broods

A number of 100 sealed broods per colony were uncovered 9 days after capping to assess *V. destructor* infection rate (%). This procedure was repeated three times for each colony, and average number of sealed broods infected with Varroa (SBIV) was used to calculate the mite infection rate in sealed broods according the below equation. The infection rate measurement was conducted two times in August.


$$Themiteinfectionratesinsealedbroods=\frac{ThenumbersofSBIV}{100}\times 100\%$$

### Reproductive property of maternal mite

The reproductive capacity of maternal mite was evaluated by analyzing the progeny of individual mother mites in sealed cells during the late pupal stage (31). Three hundred sealed cells per colony, covered for 220 h, were artificially opened, and the number of cells infected by a single mother mite (CISM) as well as the number of cells with fertile mother mites (CFM) were recorded. A mother mite was considered fertile when it co-exists with one viable adult female progeny; otherwise, it was classified as infertile. This procedure was repeated three times for each colony, and the average number was used for analysis. The sterility rate of female mites was expressed using the following formula.


$$Therateofmothermiteswithlivingoffsprings=(1-\frac{ThenumbersofCFM}{ThenumbersofCISM})\times 100\%$$

### Hygienic behavior

The hygienic behavior was assessed by the pinhold-test (32). About 100 sealed broods were deliberately pricked by a needle. Twenty-four hours later, the numbers of punctured cells with injured larvae emptied by adult bees (PCO) were recorded. This procedure was repeated three times, and the average value was used for statistical analysis. Hygienic behavior was expressed as the proportion of injured larvae that were cleaned using the following formula.


$$Hygienichehavior=\frac{ThenumbersofPCO}{Thenumbersofpuncturedcells}\times 100\%$$

### Laboratory assessment of underlying mechanisms of *F. perrara* to increase honey bee’s resistance to Varroa

#### Proboscis extension reflex (PER) test

A number of 400 worker bees at 1 day old from the same colony were randomly assigned to four sterile wooden cages (10 cm × 7 cm × 8 cm), with 100 bees per group, in the laboratory. The cages were maintained in a sterile incubator at 32°C ± 1°C and 57% ± 10% relative humidity, under dark conditions. The control group (CK) was fed a sterile 50% sucrose solution. The FP group was fed 50% sucrose solution with 1 × 10^6^ cfu/mL *F*. *perrara*. The VM group was inoculated with Varroa mites at an infective level of 10% (i.e., 10 mites per cage) and fed a sterile 50% sucrose solution. The “VM + FP” group was inoculated with Varroa mites at an infective level of 10% and fed 50% sucrose solution with 1 × 10^6^ cfu/mL *F*. *perrara*. Bees from all the treatment groups can feed *Ad libitum* for 7 days.

A number of 20 worker bees at 7 days old from either control or each treatment group (FP, VM, and VM + FP) were used for measuring the sensitivity of their olfaction by proboscis extension reflex (PER) test. Each bee was fixed in a PE tube with strips of tape so that only the two forelegs and head were free to move. After the 30-min resting period, antennae were exposed to 2 μL sucrose with three concentrations (3%, 10%, and 30% [wt/vol] in deionized water) consequentially and recorded whether the bee extended its proboscis or not. Between trials of testing different sucrose concentrations, the antennae were washed with deionized water and the bees were allowed to rest for 5 min before next concentration stimulation. The numbers of bees that responded to the same concentration sucrose were recorded. Above procedures were repeated three times for each control and treatment group.

### Assay of the relative expression levels of odorant binding proteins in antennae

Another batch of bees was reared using above methods for analyzing gene expression of OBPs that affect honey bees’ olfactory function (33, 34). At the seventh day, the antennae from 60 worker bees per group were used for RNA extraction by Trizol method (20 antenae per sample and 6 samples per group). The total RNA was immediately reverse-transcribed into cDNA using a reverse transcription Kit (TaKaRa, Dalian, China). Real-time PCR was performed using the LightCycler 480 SYBR Green RT-PCR Kit (Roche, Switzerland). The following cycling conditions were employed, including 5 min at 95°C (pre-incubation), 10 s at 95°C (denaturation), 20 s at 60°C (annealing), as well as 30 s at 72°C (extension). The primers were listed in Table 1. Relative expression levels of genes were obtained using the 2^−ΔΔCt^ method.

**TABLE 1:** Primer sequences for RT-qPCR

Genes	Primer sequences
*OBP1*-F	ATTGCTTGTTGGAGGCGTTC
*OBP1*-R	AATTATCGGAGCCGGAGGTTG
*OBP2*-F	GAACACCCTCGTCACCGTTACTTG
*OBP2*-R	TCGTCGGCGCATGGCATTATATC
*OBP4*-F	CAGGCTTCGATCTTTCCGAT
*OBP4*-R	CTCCGTAAAGTCGTCGGGTG
*OBP6*-F	TTGCGTTGCTGCTCGTCCTATTAG
*OBP6*-R	GGTGTCGTTCTTCTTGCTGCAAAC
*OBP8*-F	GCGAAACGATCGAAGAAGCAAA
*OBP8*-R	CGCCCTTCCAGTATTTCTATACAC
*OBP11*-F	CTCGTTTATGGGGAAATCAGCG
*OBP11*-R	TCCGTATTCCGTAGCTTCGAC
*OBP12*-F	AATGGCTCCGAATTGAGCAC
*OBP12*-R	GTCGTTCACGTGCAAAAACTTC
*OBP16*-F	GCGTTGGTGCAATGACACA
*OBP16*-R	TTGACTAGTGCCAGTTTCGC
*OBP21*-F	GCTGTGATACCAGTTTGTAGGA
*OBP21*-R	TCCACCATCGTCATAAGCATTG
*β-actin*-F	CCGTGATTTGACTGACTACCT
*β-actin*-R	AGTTGCCATTTCCTGTTC

### Statistical analysis and software

Statistical comparisons between two groups were analyzed using unpaired two-tailed Student’s *t*-tests in GraphPad Prism 10.1. Statistical comparisons among four treatment groups (CK, FP, VM, and FP + VM) were analyzed using ANOVA with Tukey’s multiple comparisons (GraphPad Prism 10.1.). The data are presented as the mean ± SEM. Statistical significance was determined when *P* < 0.05. Principal coordinates analysis (PCoA) of the Bray-Curtis distances was performed using the R project Vegan package (version 2.5.3) to determine significant differences between AMC and CMSC gut microbial communities.

## RESULTS

### Different Varroa infection rates and gut microbial profiles between AMC and CMSC

**Fig 1:**
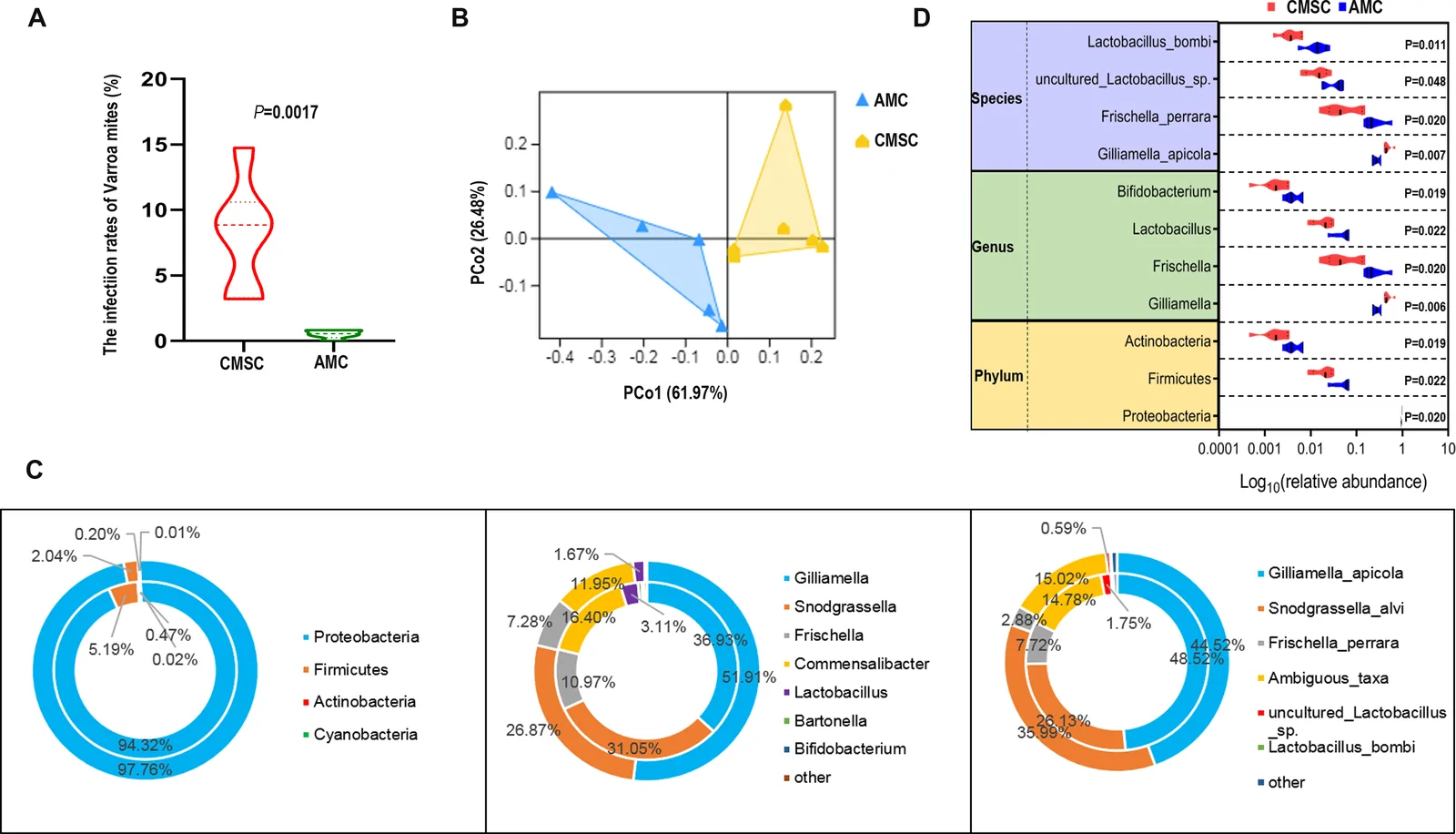
The differences in *Varroa* mite infection rates and gut bacterial taxa composition between AMC and CMSC. (A) *Varroa* mite infection rates in adult bees; (B) Principal coordinate analysis (PCoA) plot based on Bray-Curtis similarity; (C) The gut bacterial taxa of AMC (inner circle) and CMSC (outer circle). From left to right, bacteria are identified to phylum, genus, and species. (D) The differences in gut microflora between AMC and CMSC. AMC, anti-mite honey bee colonies. CMSC, conventional mites-susceptible honey bee colonies.

We found that the infection rate of Varroa mite in AMC (0.52%) was significantly lower than that in CMSC (8.04%) (*P* = 0.0017, Fig. 1A). We obtained 7,539–8,017 clean tags from the 11 samples (5 AMC and 6 CMSC) after filtering. Tag assembly and quality control yielded an average of 6,253–7,054 valid tags per sample. The average length of valid tags was 1,452.47–1,461.95 bp. Those tags were clustered into 20 OTUs. Those OTUs were mainly classified into three phyla (Proteobacteria, Firmicutes, and Actinobacteria), which accounted for more than 99.9% of all the gut bacteria (Fig. 1C). The dominant gut bacterial taxa at the genus level for AMC and CMSC included *Gilliamella*, *Snodgrassella*, *Frischella*, *Commensalibacter,* and *Lactobacillus*.

Despite the similar dominant bacterial taxa, PCoA analysis based on Bray-Curtis showed evident differences in gut bacterial community between AMC and CMSC when considering both taxonomic type and abundance (PCO1 axis representing 61.97% of overall variation) (Fig. 1B). The relative abundance of dominant gut bacterial taxa was different between AMC and CMSC. At phylum, bee guts from AMC were colonized with higher abundance of Firmicutes (*P* = 0.022) and Actinobacteria (*P* = 0.019) but less Proteobacteria compared with CMSC (*P* = 0.020, Fig. 1D). At genus, the higher abundance of *Frischella* (*P* = 0.020), *Lactobacillus* (*P* = 0.022), and *Bifidobacterium* (*P* = 0.019) was detected in AMC, but abundance of *Gilliamella* (*P* = 0.006, Fig. 1D) was higher in CMSC. At the species, AMC possessed higher abundance of *F. perrara* (*P* = 0.020), *Lactobacillus bombi* (*P* = 0.011), and *uncultured Lactobacillus* spp. (*P* = 0.048), but lower *Gilliamella* (*P* = 0.007) than CMSC (Fig. 1D).

### Improved Varroa-tolerance traits of susceptible colonies fed with *F. perrara* in field experiments

**Fig 2:**
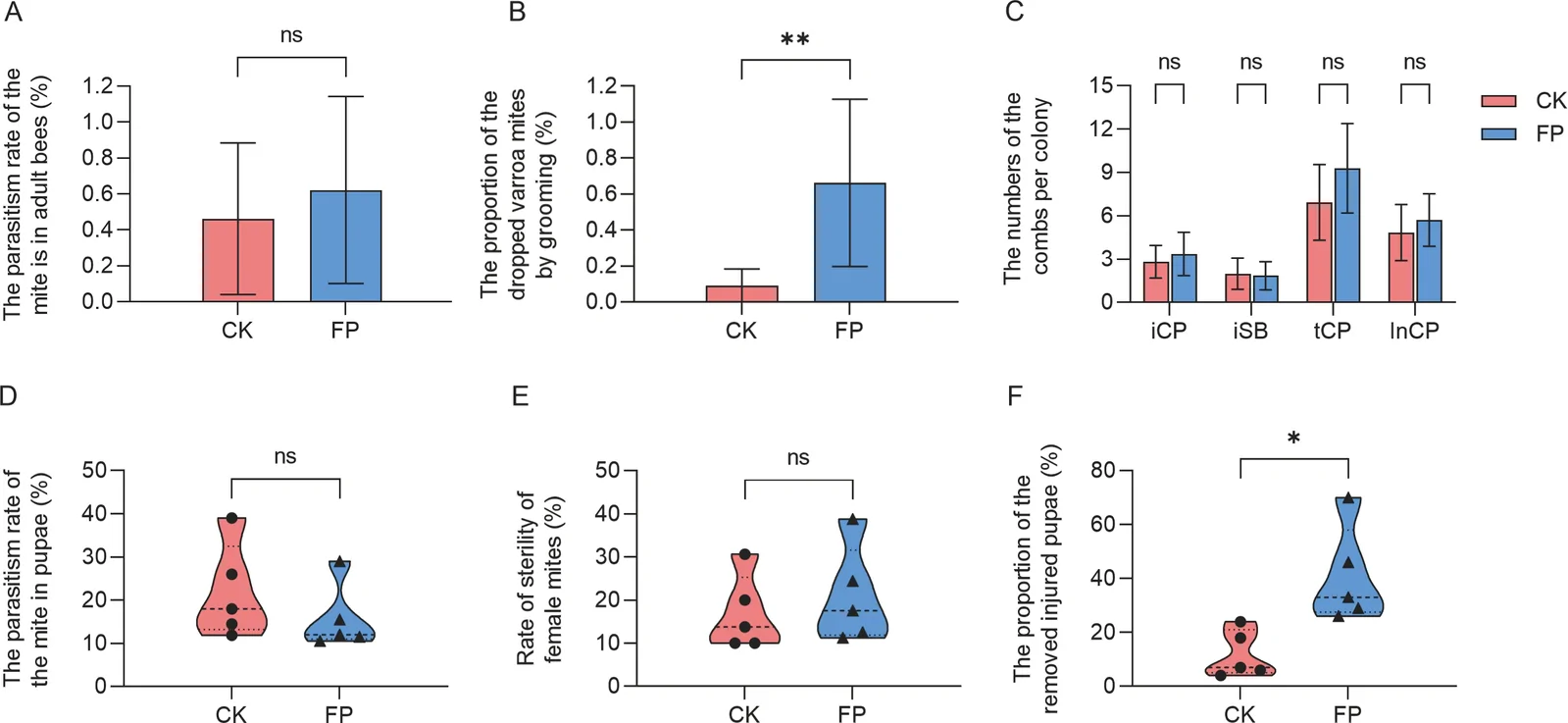
Varroa-tolerant traits of bee colonies. CK, control colonies fed with 50% sucrose. FP, experimental colonies fed with 1 × 10^6^ cfu/mL *F*. *perrara* in 50% sucrose. iCP, initial colony population. iSB, initial sealed brood. tCP, terminal colony population. InCP, increased colony population = tCP iCP. *P < 0.05, **P < 0.01; ns, no significant difference.

Since *F. perrara* has not been tested for its effect in Varroa tolerance in any other previous studies, this bacterium was assessed in our study. Feeding *F. perrara* did not reduce the *Varroa* infection rate of adult bees (Fig. 2A), but significantly increased the proportion of the dropped Varroa mite (*P* = 0.001) (Fig. 2B). Administration of *F. perrara* did not change brood and adult bee population (Fig. 2C). Feeding *F. perrara* did not significantly reduce the infection rate of Varroa mites in sealed brood (*P* = 0.332, Fig. 2D) or the rate of living offsprings (*P* = 0.547, Fig. 2E); however, this bacterial treatment increased honey bees’ activity to clean injured pupae (*P* = 0.012) (Fig. 2F).

### Improved the olfactory ability of work bees fed with *F. perrara* in lab experiments

**Fig 3:**
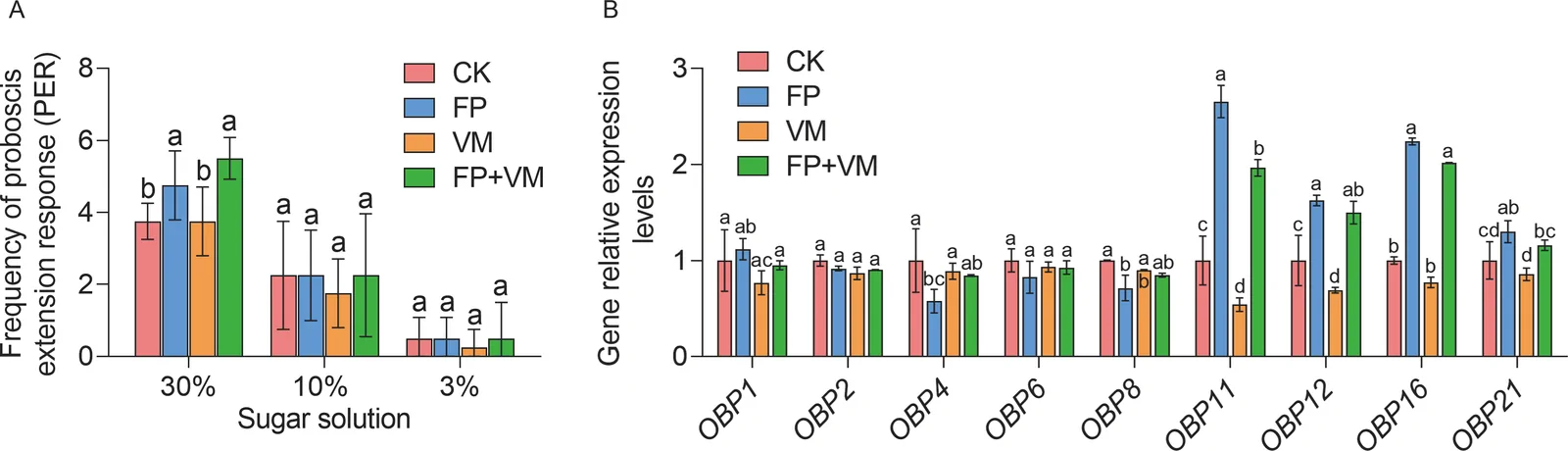
Effects of *F. perrara* on bees’ olfaction sensitivity through PER tests and OBP gene expression. Bees were reared in four groups (CK, FP, VM, and VM + FP) before PER tests to assess bees’ responses to sugar solutions of three concentrations. CK, control bees fed with 50% sucrose. FP, experimental bees fed with 1 × 10^6^ cfu/mL *F*. *perrara* in 50% sucrose. VM, bees inoculated with Varroa mites at an infective level of 10% and fed sterile 50% sucrose solution. VM + FP, bees inoculated with Varroa mites at an infective level of 10% and fed 1 × 10^6^ cfu/mL *F*. *perrara* in 50% sucrose solution. Bars with different lowercase letters represent significant differences (*P* < 0.05).

The effects of *F. perrara* on the olfactory sensitivity of bees to different concentrations of sugar solution were evaluated by proboscis extension reflex (PER) test (Fig. 3). Compared with control bees or bees infected with Varroa mite, those fed with *F. perrara* can significantly increase PER frequency of honey bees to 30% sugar solution (*P* = 0.02, Tukey multiple comparisons, Fig. 3A). When fed with 10% and 3% syrup, *F. perrara* did not significantly change PER frequency (10% syrup: *P* = 0.94; 3% syrup: *P* = 0.94). Feeding *F. perrara* can significantly upregulate the expression of four odorant binding protein (including OBP11, OBP12, OBP16, and OBP21; *P* < 0.0001 with Tukey multiple comparisons) (Fig. 3B).

## DISCUSSION

In our study, the lower Varroa infection rate of AMC surviving from the acaracide-free apiary suggests elevated Varroa-tolerance. We found significant increases in the proportion of *Frischella*, *Lactobacillus,* and *Bifidobacterium* when comparing the gut bacteria between untreated surviving honey bees (AMC) and treated conventional Varroa-susceptible honey bees (CMSC). The changes in the abundance of those gut symbiotic bacteria suggest potential active involvement of symbiotic bacteria in Varroa suppression. The naturally resident bacterial community in the gastrointestinal tract is found to affect host health such parasitic or disease infection for various insects except for honey bees. For example, a maternally transmitted bacterium, *Spiroplasma* spp., protects fruit flies against parasitic nematodes, in both the laboratory and field conditions (35). Colonization of the mosquito midgut by symbiotic bacteria *Serratia marcescens* renders the mosquito resistance to *Plasmodium berghei* infection through activation of the mosquito immune system (36). Socially transmitted gut bacteria protect bumblebees against a widespread and highly virulent intestinal parasites (*Crithidia bombi*) (17). A healthy gut bacterial community in honey bees protects them from infectious bacteria (*Serratia marcescens*) and fungus (*Beauveria bassiana* and *Aspergillus flavus*) (37, 38).

Among those bacteria genera being significantly different between AMC and CMSC in our study, *Lactobacillus* and *Bifidobacterium* have been proven to be effective in reducing Varroa infestation (26, 27). So, we did not choose to test the effects of feeding *Lactobacillus* and *Bifidobacterium* to colonies in our study again. The correlation of *Frischella* with lower Varroa infestation was validated in our study, confirming their true impact on Varroa control. We chose to test our hypothesis that *F. perrara* is responsible for developing tolerance to Varroa. *F. perrara* has been found to enhance honey bees’ immunity to diseases by its involvement in melanization (39, 40). When administering *F. perrara* to Varroa-susceptible colonies in the field, the increasing number of fallen Varroa as well as more injured pupae to be removed provides evidence of bees developing tolerance to Varroa. However, *F. perrara* cannot compromise Varroa health in capped brood, and Varroa may successfully camouflage through their chemical mimicry to brood (41). Our field results suggest *F. perrara* may regulate bees’ hygienic and grooming behavior to increase their ability to remove phoretic Varroa, which two behaviors are major characteristics of Varroa tolerance (42–44). Although we found more dropped mites on the bottom board, the phoretic mites as well as reproductive mites in capped brood did not decrease potentially due to too short monitoring time to observe treatment effects.

The mechanism of symbionts to suppress parasitic infections remains unclear (23). Gut microbes can act as a chemo-olfactory recognition signal to change an animal’s behavior (45). We hypothesized that *F. perrara* may enhance honey bees’ olfaction function. The laboratory PER experiment showed that *F. perrara* did improve bees’ olfactory sensitivity, which may be driven by increased expression of OBPs (OBP11, OPB12, OBP16, and OPB21). However, the other study did not find elevated expression of OBPs in bee’s head when they were collected from Varroa-tolerant colonies (46). Since the experimental designs are different between the previous and the current study, the reason to observe different trends of OBP gene expression remains unclear. The molecular mechanism of *F. perrara* to affect bees’ olfactory and tolerance to Varroa requires further exploration beyond our work.

In summary, our genetic sequencing and gene expression study indicated that Vorroa-tolerant trait of western honey bees is correlated with physiological factors, i.e., intestinal microbial community. Through the bacterial feeding experiments in the field and lab, we found solid causal evidence that *F. perrara* can strengthen honey bees’ tolerance to Varroa. We suggest the underlying mechanisms are to enhance hygiene and grooming behaviors by elevating bees’ olfactory sensitivity. However, *F. perrara* did not change all hygienic parameters, and the efficacy of *F. perrara* treatment in fields should be further improved before this method can become an effective Varroa control technique. One limitation of our study is that we did not test if the bacterial community as a whole affects Varroa tolerance, which is an important question to answer in future.

## Data Availability

The raw ^16^S rDNA sequencing data generated in this study were submitted to the NCBI Sequence Read Archive (SRA) and can be accessed publicly under the accession number PRJNA1206749(47).

## References

[1] Hasegawa N, Techer MA, Adjlane N, Al-Hissnawi MS, Antúnez K, Beaurepaire A, Christmon K, Delatte H, Dukku UH, Eliash N, El-Niweiri MAA, Esnault O, Evans JD, Haddad NJ, Locke B, Muñoz I, Noël G, Panziera D, Roberts JMK, De la Rúa P, Shebl MA, Stanimirovic Z, Rasmussen DA, Mikheyev AS. 2023. Evolutionarily diverse origins of deformed wing viruses in western honey bees. Proc Natl Acad Sci USA 120:e2301258120. doi:10.1073/pnas.2301258120 37339224 PMC10293827

[2] Kruitwagen A, van Langevelde F, van Dooremalen C, Blacquière T. 2017. Naturally selected honey bee ( *Apis mellifera* ) colonies resistant to *Varroa destructor* do not groom more intensively. J Apic Res 56:354–365. doi:10.1080/00218839.2017.1329797

[3] Brook G. 2025. Varroa treatment-free colony losses in the European honey bee (*Apis mellifera):* a review of published literature. J Apic Res:1–10. doi:10.1080/00218839.2025.2514936

[4] Rosenkranz P, Aumeier P, Ziegelmann B. 2010. Biology and control of *Varroa destructor*. J Invertebr Pathol 103 Suppl 1:S96–119. doi:10.1016/j.jip.2009.07.016 19909970

[5] Beaurepaire AL, Krieger KJ, Moritz RFA. 2017. Seasonal cycle of inbreeding and recombination of the parasitic mite *Varroa* destructor in honeybee colonies and its implications for the selection of acaricide resistance. Infect Genet Evol 50:49–54. doi:10.1016/j.meegid.2017.02.011 28216419

[6] Broeckx BJG, De Smet L, Blacquière T, Maebe K, Khalenkow M, Van Poucke M, Dahle B, Neumann P, Bach Nguyen K, Smagghe G, Deforce D, Van Nieuwerburgh F, Peelman L, de Graaf DC. 2019. Honey bee predisposition of resistance to ubiquitous mite infestations. Sci Rep 9:7794. doi:10.1038/s41598-019-44254-8 31127129 PMC6534585

[7] Fries I, Imdorf A, Rosenkranz P. 2006. Survival of mite infested (*Varroa destructor*) honey bee (*Apis mellifera*) colonies in a Nordic climate. Apidologie (Celle) 37:564–570. doi:10.1051/apido:2006031

[8] Guzman-Novoa E, Corona M, Alburaki M, Reynaldi FJ, Invernizzi C, Fernández de Landa G, Maggi M. 2024. Honey bee populations surviving Varroa destructor parasitism in Latin America and their mechanisms of resistance. Front Ecol Evol 12:1434490. doi:10.3389/fevo.2024.1434490

[9] Le Conte Y, Vaublanc G, Crauser D, Jeanne F, Rousselle J-C, Bécard J-M. 2007. Honey bee colonies that have survived Varroa destructor. Apidologie (Celle) 38:566–572. doi:10.1051/apido:2007040

[10] Le Conte Y, Meixner MD, Brandt A, Carreck NL, Costa C, Mondet F, Büchler R. 2020. Geographical distribution and selection of European honey bees resistant to *Varroa destructor*. Insects 11:873. doi:10.3390/insects11120873 33302465 PMC7764010

[11] Seeley TD. 2007. Honey bees of the Arnot Forest: a population of feral colonies persisting with *Varroa destructor* in the northeastern United States. Apidologie (Celle) 38:19–29. doi:10.1051/apido:2006055

[12] Grindrod I, Martin SJ. 2021. Parallel evolution of *Varroa* resistance in honey bees: a common mechanism across continents? Proc Biol Sci 288:20211375. doi:10.1098/rspb.2021.1375 34344183 PMC8334839

[13] Ibrahim A, Spivak M. 2006. The relationship between hygienic behavior and suppression of mite reproduction as honey bee (*Apis mellifera* ) mechanisms of resistance to *Varroa destructor*. Apidologie (Celle) 37:31–40. doi:10.1051/apido:2005052

[14] Mondragón L, Spivak M, Vandame R. 2005. A multifactorial study of the resistance of honeybees *Apis mellifera* to the mite *Varroa destructor* over one year in Mexico. Apidologie (Celle) 36:345–358. doi:10.1051/apido:2005022

[15] Locke B, Conte YL, Crauser D, Fries I. 2012. Host adaptations reduce the reproductive success of Varroa destructor in two distinct European honey bee populations. Ecol Evol 2:1144–1150. doi:10.1002/ece3.248 22833790 PMC3402190

[16] Engel P, Martinson VG, Moran NA. 2012. Functional diversity within the simple gut microbiota of the honey bee. Proc Natl Acad Sci USA 109:11002–11007. doi:10.1073/pnas.1202970109 22711827 PMC3390884

[17] Koch H, Schmid-Hempel P. 2011. Socially transmitted gut microbiota protect bumble bees against an intestinal parasite. Proc Natl Acad Sci USA 108:19288–19292. doi:10.1073/pnas.1110474108 22084077 PMC3228419

[18] Leonard SP, Powell JE, Perutka J, Geng P, Heckmann LC, Horak RD, Davies BW, Ellington AD, Barrick JE, Moran NA. 2020. Engineered symbionts activate honey bee immunity and limit pathogens. Science 367:573–576. doi:10.1126/science.aax9039 32001655 PMC7556694

[19] Babendreier D, Joller D, Romeis J, Bigler F, Widmer F. 2007. Bacterial community structures in honeybee intestines and their response to two insecticidal proteins. FEMS Microbiol Ecol 59:600–610. doi:10.1111/j.1574-6941.2006.00249.x 17381517

[20] Jeyaprakash A, Hoy MA, Allsopp MH. 2003. Bacterial diversity in worker adults of *Apis mellifera* capensis and *Apis mellifera* scutellata (Insecta: Hymenoptera) assessed using 16S rRNA sequences. J Invertebr Pathol 84:96–103. doi:10.1016/j.jip.2003.08.007 14615218

[21] Moran NA, Hansen AK, Powell JE, Sabree ZL. 2012. Distinctive gut microbiota of honey bees assessed using deep sampling from individual worker bees. PLoS One 7:e36393. doi:10.1371/journal.pone.0036393 22558460 PMC3338667

[22] Koch H, Schmid‐Hempel P. 2012. Gut microbiota instead of host genotype drive the specificity in the interaction of a natural host‐parasite system. Ecol Lett 15:1095–1103. doi:10.1111/j.1461-0248.2012.01831.x 22765311

[23] Motta EVS, Moran NA. 2024. The honeybee microbiota and its impact on health and disease. Nat Rev Microbiol 22:122–137. doi:10.1038/s41579-023-00990-3 38049554 PMC10998682

[24] Hubert J, Bicianova M, Ledvinka O, Kamler M, Lester PJ, Nesvorna M, Kopecky J, Erban T. 2017. Changes in the bacteriome of honey bees associated with the parasite Varroa destructor, and pathogens *Nosema* and *Lotmaria passim*. Microb Ecol 73:685–698. doi:10.1007/s00248-016-0869-7 27730366

[25] Kim M, Kim WJ, Park SJ. 2022. Gut microbiota analysis of the western honeybee (*Apis mellifera* L.) infested with the mite *Varroa destructor* reveals altered bacterial and archaeal community. Microbiology. doi:10.1101/2022.04.20.488909

[26] Audisio MC, Sabaté DC, Benítez-Ahrendts MR. 2015. Effect of Lactobacillus johnsonii CRL1647 on different parameters of honeybee colonies and bacterial populations of the bee gut. Benef Microbes 6:687–695. doi:10.3920/BM2014.0155 25809216

[27] Saccà ML, Lodesani M. 2020. Isolation of bacterial microbiota associated to honey bees and evaluation of potential biocontrol agents of *Varroa destructor* Benef Microbes 11:641–654. doi:10.3920/BM2019.0164 33124896

[28] Dietemann V, Nazzi F, Martin SJ, Anderson DL, Locke B, Delaplane KS, Wauquiez Q, Tannahill C, Frey E, Ziegelmann B, Rosenkranz P. 2013. Standard methods for varroa research. J Apic Res 52:1–54.

[29] Yu J, Zhang W, Chi X, Chen W, Li Z, Wang Y, Liu Z, Wang H, Xu B. 2022. The dietary arachidonic acid improved growth and immunity of honey bee (*Apis mellifera ligustica* ). Bull Entomol Res 112:261–270. doi:10.1017/S0007485321000821 34622750

[30] Wang H, Liu C, Liu Z, Wang Y, Ma L, Xu B. 2020. The different dietary sugars modulate the composition of the gut microbiota in honeybee during overwintering. BMC Microbiol 20:61. doi:10.1186/s12866-020-01726-6 32183692 PMC7076957

[31] Calderón RA, Zamora LG, Van Veen JW, Quesada MV. 2007. A comparison of the reproductive ability of *Varroa destructor* (Mesostigmata:Varroidae) in worker and drone brood of Africanized honey bees (*Apis mellifera*). Exp Appl Acarol 43:25–32. doi:10.1007/s10493-007-9102-1 17828439

[32] Spivak M, Downey DL. 1998. Field assays for hygienic behavior in honey bees (*Hymenoptera: Apidae*). J Apic Res 91:64–70. doi:10.1093/jee/91.1.64

[33] Liu F, Lai Y, Wu L, Li Q, Lei L, Yin W, Zhang Y, Huang ZY, Zhao H. 2025. AmelOBP4: an antenna-specific odor-binding protein gene required for olfactory behavior in the honey bee (Apis mellifera). Front Zool 22:2. doi:10.1186/s12983-024-00554-y 39810219 PMC11731170

[34] McAfee A. 2018. Towards defining the molecular mechanism of hygienic behaviour in honey bees (Apis mellifera) Doctoral dissertation, University of British Columbia

[35] Jaenike J, Unckless R, Cockburn SN, Boelio LM, Perlman SJ. 2010. Adaptation via symbiosis: recent spread of a *Drosophila defensive* symbiont. Science 329:212–215. doi:10.1126/science.1188235 20616278

[36] Bai L, Wang L, Vega-Rodríguez J, Wang G, Wang S. 2019. A gut symbiotic bacterium *Serratia* marcescens renders mosquito resistance to *Plasmodium* infection through activation of mosquito immune responses. Front Microbiol 10:1580. doi:10.3389/fmicb.2019.01580 31379768 PMC6657657

[37] Miller DL, Smith EA, Newton ILG. 2021. A bacterial symbiont protects honey bees from fungal disease. mBio 12:e0050321. doi:10.1128/mBio.00503-21 34101488 PMC8262860

[38] Steele MI, Motta EVS, Gattu T, Martinez D, Moran NA. 2021. The gut microbiota protects bees from invasion by a bacterial pathogen. Microbiol Spectr 9:e00394–21. doi:10.1128/Spectrum.00394-21 34523998 PMC8557934

[39] Emery O, Schmidt K, Engel P. 2017. Immune system stimulation by the gut symbiont Frischella perrara in the honey bee (Apis mellifera). Mol Ecol 26:2576–2590. doi:10.1111/mec.14058 28207182

[40] Engel P, Bartlett KD, Moran NA. 2015. The bacterium *Frischella perrara* causes scab formation in the gut of its honeybee host. mBio 6:e00193–15. doi:10.1128/mBio.00193-15 25991680 PMC4442143

[41] Kather R, Drijfhout FP, Shemilt S, Martin SJ. 2015. Evidence for passive chemical camouflage in the parasitic mite Varroa destructor. J Chem Ecol 41:178–186. doi:10.1007/s10886-015-0548-z 25620373

[42] Arechavaleta-Velasco ME, Guzmn-Novoa E. 2001. Relative effect of four characteristics that restrain the population growth of the mite *Varroa destructor* in honey bee (*Apis mellifera* ) colonies. Apidologie (Celle) 32:157–174. doi:10.1051/apido:2001121

[43] Kurze C, Routtu J, Moritz RFA. 2016. Parasite resistance and tolerance in honeybees at the individual and social level. Zoology (Jena) 119:290–297. doi:10.1016/j.zool.2016.03.007 27106014

[44] Nganso BT, Fombong AT, Yusuf AA, Pirk CWW, Stuhl C, Torto B. 2017. Hygienic and grooming behaviors in African and European honeybees-New damage categories in *Varroa destructor*. PLoS One 12:e0179329. doi:10.1371/journal.pone.0179329 28622341 PMC5473549

[45] Ezenwa VO, Williams AE. 2014. Microbes and animal olfactory communication: where do we go from here? Bioessays 36:847–854. doi:10.1002/bies.201400016 24986361

[46] Russo RM, Pietronave H, Conte CA, Liendo MC, Basilio A, Lanzavecchia SB, Scannapieco AC. 2024. Stimulus-specific gene expression profiles associated with grooming behavior and Varroa destructor resistance in honey bees. Frontiers in Bee Science 2:1441317.

[47] Wang H, Niu X, Lei L, Zhou T, Zhang G, Xu B. 2022. Data from “the project to evaluate if the symbiotic bacteria Frischella perrara in honey bees mitigate *Varroa mite* infection. GenBank. Available from: https://www.ncbi.nlm.nih.gov/Traces/study/?acc=PRJNA1206749&o=acc_s%3Aa 10.1128/spectrum.00960-26 PMC13340150 42294715

